# Exogenous exposures shape genetic predisposition to lipids, Alzheimer’s, and coronary heart disease in the MLXIPL gene locus

**DOI:** 10.18632/aging.204665

**Published:** 2023-04-18

**Authors:** Yury Loika, Elena Loiko, Fan Feng, Eric Stallard, Anatoliy I. Yashin, Konstantin Arbeev, Allison L. Kuipers, Mary F. Feitosa, Michael A. Province, Alexander M. Kulminski

**Affiliations:** 1 Biodemography of Aging Research Unit, Social Science Research Institute, Duke University, Durham, NC 27708, USA; 2 Department of Epidemiology, University of Pittsburgh, Pittsburgh, PA 15260, USA; 3 Division of Statistical Genomics, Department of Genetics, Washington University School of Medicine, St Louis, MO 63110, USA

**Keywords:** *MLXIPL*, lipids, triglycerides, coronary heart disease, Alzheimer's disease

## Abstract

Associations of single nucleotide polymorphisms (SNPs) of the *MLXIPL* lipid gene with Alzheimer’s (AD) and coronary heart disease (CHD) and potentially causal mediation effects of their risk factors, high-density lipoprotein cholesterol (HDL-C) and triglycerides (TG), were examined in two samples of European ancestry from the US (22,712 individuals 587/2,608 AD/CHD cases) and the UK Biobank (UKB) (232,341 individuals; 809/15,269 AD/CHD cases). Our results suggest that these associations can be regulated by several biological mechanisms and shaped by exogenous exposures. Two patterns of associations (represented by rs17145750 and rs6967028) were identified. Minor alleles of rs17145750 and rs6967028 demonstrated primary (secondary) association with high TG (lower HDL-C) and high HDL-C (lower TG) levels, respectively. The primary association explained ~50% of the secondary one suggesting partly independent mechanisms of TG and HDL-C regulation. The magnitude of the association of rs17145750 with HDL-C was significantly higher in the US vs. UKB sample and likely related to differences in exogenous exposures in the two countries. rs17145750 demonstrated a significant detrimental indirect effect through TG on AD risk in the UKB only (β_IE_ = 0.015, p_IE_ = 1.9 × 10^−3^), which suggests protective effects of high TG levels against AD, likely shaped by exogenous exposures. Also, rs17145750 demonstrated significant protective indirect effects through TG and HDL-C in the associations with CHD in both samples. In contrast, rs6967028 demonstrated an adverse mediation effect through HDL-C on CHD risk in the US sample only (β_IE_ = 0.019, p_IE_ = 8.6 × 10^−4^). This trade-off suggests different roles of triglyceride mediated mechanisms in the pathogenesis of AD and CHD.

## INTRODUCTION

The number of people aged 65 years and over is projected to reach 1.5 billion worldwide in the next three decades, comprising 16% of the total population [1]. Coronary heart disease (CHD) and Alzheimer’s disease (AD) will remain as leading causes of mortality and of disability related to cardiovascular system health and brain health [2]. Recent findings suggest that neurodegenerative and cardiovascular diseases may have overlapping etiologies [3, 4], implying that the health of the brain, the heart, and blood vessels are closely connected. Increasing age is a major non-modifiable risk factor shared by all age-related diseases, including AD and CHD. However, growing evidence suggests that there is a set of modifiable factors shared between AD and CHD [3], including diabetes, obesity, hypertension, hypercholesterolemia, atherosclerosis, and lack of exercise.

Some of these modifiable factors can be common risk factors with homogeneous effects; others can demonstrate complex heterogeneous patterns that might change the effect-direction with age and/or in different populations. Currently, there is no consensus about effects of different modifiable risk factors in predisposition to AD and about changes of their effects during aging. Some studies have shown that vascular atherosclerotic pathology, a hallmark of cardiovascular disease, is also present in individuals with AD [5]. Other studies demonstrated that high levels of several modifiable factors, such as body mass index (BMI), blood pressure (BP), and blood cholesterol, at midlife are associated with increased risk of late-onset AD, while their high levels in later life can be protective in some populations [6–9]. Moreover, when measured at midlife (55 years old), a high level of CHD risk — e.g., using the Framingham CHD risk score, [10] which includes information on age, total cholesterol (TC), high-density lipoprotein cholesterol (HDL-C), blood pressure (BP), smoking status, and diabetes (T2D) — was concurrently associated with lower levels of general cognitive performance and memory. However, when measured in later-life (70+), an elevated CHD risk score was concurrently associated with better cognitive performance and memory [11]. These findings suggest a more complex pattern of the effects of CHD risk factors in predisposition to CHD and AD with possible tradeoff contributions.

Altered lipoprotein synthesis and altered lipid and glucose metabolism might be common mechanisms of pathological action of major genetic risk factors of AD and CHD. MLXIPL (MLX-interacting-protein-like; or ChREBP, carbohydrate response element binding protein) regulates glycolysis and lipogenesis at the transcription level [12] and, therefore, may contribute to the development and progression of both these diseases. Activation of glycolysis and lipogenesis pathways by the *MLXIPL* gene results in the synthesis of triglycerides (TG). The genome-wide significant association of single nucleotide polymorphisms (SNPs) from the *MLXIPL* gene locus with TG levels was previously reported [13–16]. Studies have also demonstrated associations of *MLXIPL* genetic markers with levels of serum very-low density lipoprotein (VLDL) [17] and HDL-C [18, 19]. *MLXIPL* has been identified as a risk factor for diseases such as hyperglycemia [20], diabetes [21], non-alcoholic fatty liver diseases [22], and CHD [23–26].

Previous studies demonstrated contradictory results about the associations of circulating lipids in serum with AD risk [4, 27]. Additionally, there have been no reported associations of SNPs from the *MLXIPL* gene locus with AD, so the mechanisms that may underlie any causal connections between *MLXIPL*, lipids, AD, and CHD, remain poorly understood. Previously, a Mendelian randomization (MR) study [28], which tested causal effects of HDL-C, LDL-C, and TG on CHD and AD risk, showed that (a) higher levels of HDL-C and TG and lower levels of LDL-C were associated with decreased AD risk, while (b) high levels of HDL-C and lower levels of TG and LDL-C were associated with decreased CHD risk. However, those results were not statistically significant at the conventional 0.05 level.

In this paper, we use an alternative approach and greater sample size to address similar questions. We identified and examined the connections between the outlined traits by utilizing causal mediation analyses [29–33] as well as unconditional and conditional univariate regressions. The analyses leverage longitudinal information on lipids assessed at different ages prior to the onset of AD and CHD in two samples of European ancestry including (i) 22,712 individuals (587/2,608 AD/CHD cases) from seven US cohorts and (ii) 232,341 individuals (809/15,269 AD/CHD cases) from the UK Biobank (UKB). We describe the complex structure of lipid regulation by SNPs from the *MLXIPL* gene locus, the mutual mediation roles of triglycerides and HDL-C in the associations of these SNPs with CHD, and the role of triglycerides as a potential mediator in the associations of these SNPs with AD. These analyses may help dissect factor-specific heterogeneity — related to the effects of exogenous exposures and plausible biological mechanisms — in the associations between lipids, AD, and CHD, and suggest potential causal relationships.

## RESULTS

### Study overview

The analyses were performed for AD, CHD, and four continuous phenotypes of lipids (HDL-C, TG, LDL-C, and TC) in individuals of European ancestry, for men and women combined, from seven independent US cohorts and, independently, in UK Biobank (Supplementary Table 1, Supplementary Data). The results were meta-analyzed across US cohorts and compared with respective results from the UK Biobank. In US cohorts, as not all phenotypes were available in each study, we used two samples, one sample for AD and the other one for non-AD analyses, to maximize their size and increase the power. The analyses on predisposition to AD were performed using the sample of 11,112 individuals including 587 AD-affected individuals from the CHS, FHS_C1, FHS_C2, and LLFS cohorts (Supplementary Table 1, Supplementary Data). The analyses with CHD and all continuous phenotypes in the US cohorts were performed in a sample of 22,712 participants, which included 2,608 incident CHD cases from ARIC, CHS, FHS_C1, FHS_C2, MESA, WHI, and LLFS (Supplementary Table 1, Supplementary Data). Separately, the analyses were performed in the UK Biobank (UKB) for 232,341 individuals of European ancestry (Supplementary Table 1, Supplementary Data) including 809 AD and 15,269 CHD cases. Analyses for lipid traits were performed by using information from all available examinations in the US based cohorts (95,826 person-observations) and information from one examination only in the UKB. Lipid measurements at last available examination or at an examination preceding the disease onset were used in the mediation analyses.

Four SNPs were selected to represent three sets of independent SNPs defined by linkage disequilibrium (see Methods) in the *MLXIPL* gene locus: (i) rs1051921, and rs17145750, (ii) rs6967028, and (iii) rs11760752 (Supplementary Table 2). Minor allele frequency for the selected SNPs (Supplementary Table 2) varied between 8.36% (rs6967028) in the FHS_C1 to 21.9% (rs1051921) in the WHI cohort. The selected SNPs were in a weak linkage disequilibrium (LD), r^2^ < 6%, except rs1051921 and rs17145750 (73% < r^2^ < 80%) (Supplementary Table 3). The correlation between the lipid traits (Supplementary Table 4) varied from −48% (TG–HDL-C correlation in the WHI cohort) to 95% (TC–LDL-C correlation in the UKB). Weak to moderate correlation was observed between the lipid traits and CHD (Supplementary Table 4), ranging from −18% (CHD–HDL-C correlation in the ARIC cohort) to 11% (CHD–TG correlation in the CHS cohort). Much weaker correlations were observed between the lipid traits and AD (Supplementary Table 4), which ranged −4% (AD–HDL-C correlation in the LLFS cohort) to 7% (AD–HDL-C correlation in the FHS_C1 cohort).

### Two sets of SNPs demonstrated different patterns of associations with lipids

**Figure 1:**
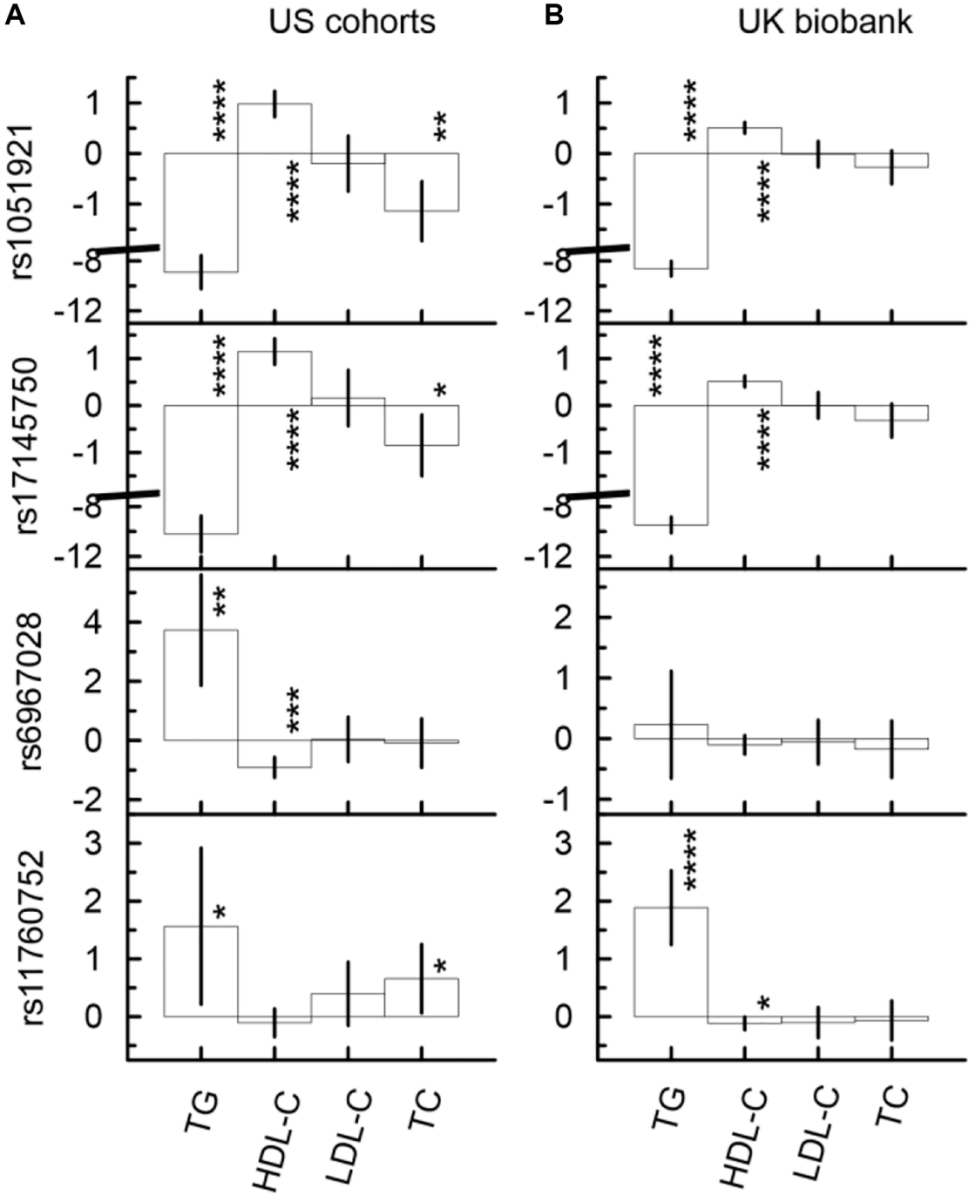
Univariate unconditional associations of four SNPs from the *MLXIPL* gene with four continuous traits, TG (mg/dL), HDL-C (mg/dL), LDL-C (mg/dL), and LDL-C (mg/dL) in two samples drawn from US cohorts (**A**) and UK biobank (**B**). Two set of SNPs with different patterns of the associations are observed. The first (second) set, which include rs1051921 and rs17145750 (rs6967028 and rs11760752) demonstrate significant associations with higher (lower) levels of TG and lower (higher) levels of HDL-C. The Y-axis shows the effect sizes (beta) of the associations of respective SNPs with the outcomes depicted on the X axis. The X-axis shows the outcome variable used in the model. The vertical solid lines indicate 95% confidence intervals (CIs). Asterisks indicate different levels of significance, i.e., ^*^5 × 10^−4^ ≤ *p* < 0.05; ^**^5 × 10^−6^ ≤ *p* < 5 × 10^−4^; ^***^5 × 10^−8^ ≤ *p* < 5 × 10^−6^; ^****^p < 5 × 10^−8^.

The univariate unconditional analysis, which included longitudinal measurements, identified two sets and related patterns in the associations of SNPs from the *MLXIPL* gene locus with lipids. The difference between these patterns was determined by the strength and direction of the associations (i.e., beta coefficients). The first (second) pattern was characterized by significantly lower (higher) levels of TG and higher (lower) levels of HDL-C in carriers of their minor alleles (Figure 1, Supplementary Table 5, Supplementary Figure 1). Two SNPs, rs1051921 and rs17145750, represented the first pattern and were within the same LD block. Also, these SNPs demonstrated consistent significant association with lower levels of TC in their minor allele carriers. (Note, the association of rs17145750 with TC was non-significant after correction for multiple testing, i.e., *p*-value < *p_MT_* = 1.79 × 10^−3^.) rs6967028 represents the second pattern (Supplementary Figure 1). The associations of rs6967028, rs1051921, and rs17145750 with TG and HDL-C remained significant in the models that included any pair of these SNPs (even after correction for multiple testing), which demonstrated independence of their associations. The pattern of associations for rs11760752 was similar to that of the second one but was not independent of rs1051921 or rs17145750 (Supplementary Table 5B).

The univariate conditional analysis provided further evidence on the differences between the first and the second sets of SNPs. The association with TG (HDL-C) was the leading association in the first (second) set. For instance (Supplementary Table 5A), in the US meta-analysis the magnitude of the rs1051921 association with HDL-C (*β* = 0.98, *p*-value = 3.15 × 10^−14^) decreased by 52% after adjustment by TG (*β* = 0.47, *p*-value = 6.84 × 10^−5^), while its associations with TG (*β* = −8.90, *p*-value = 8.28 × 10^−39^) decreased by 23% after adjustment by HDL-C (*β* = −6.89, *p*-value = 6.39 × 10^−30^). Additionally, the association with TC (*β* = −1.14, *p*-value = 1.56 × 10^−4^) was completely explained by TG (*β* = 0.04, *p*-value = 8.80 × 10^−1^ after adjustment by TG). In contrast, the magnitude of the rs6967028 association with TG (*β* = 3.73, *p*-value = 9.42 × 10^−5^) decreased by 53% after adjustment by HDL-C (*β* = 1.76, *p*-value = 3.62 × 10^−2^), which was non-significant after correction for multiple-testing (*p_MT_* = 1.79 × 10^−3^), while its association with HDL-C (*β* = −0.91, *p*-value = 2.15 × 10^−7^) decreased only by 29% in magnitude after adjustment by TG (*β* = −0.65, *p*-value = 5.22 × 10^−5^) and remained statistically significant even after correction for multiple testing.

In the UKB (Figure 1 and Supplementary Table 5C, 5D), the same pattern of association was observed for SNPs from the first set and for the rs11760752 associations with TG and HDL-C. For rs6967028, the sign directions of all associations were consistent with those observed in the US meta-analysis, but they did not attain statistical significance.

### Minor allele of rs17145750 was associated with significantly higher AD risk in the UK Biobank

**Figure 2:**
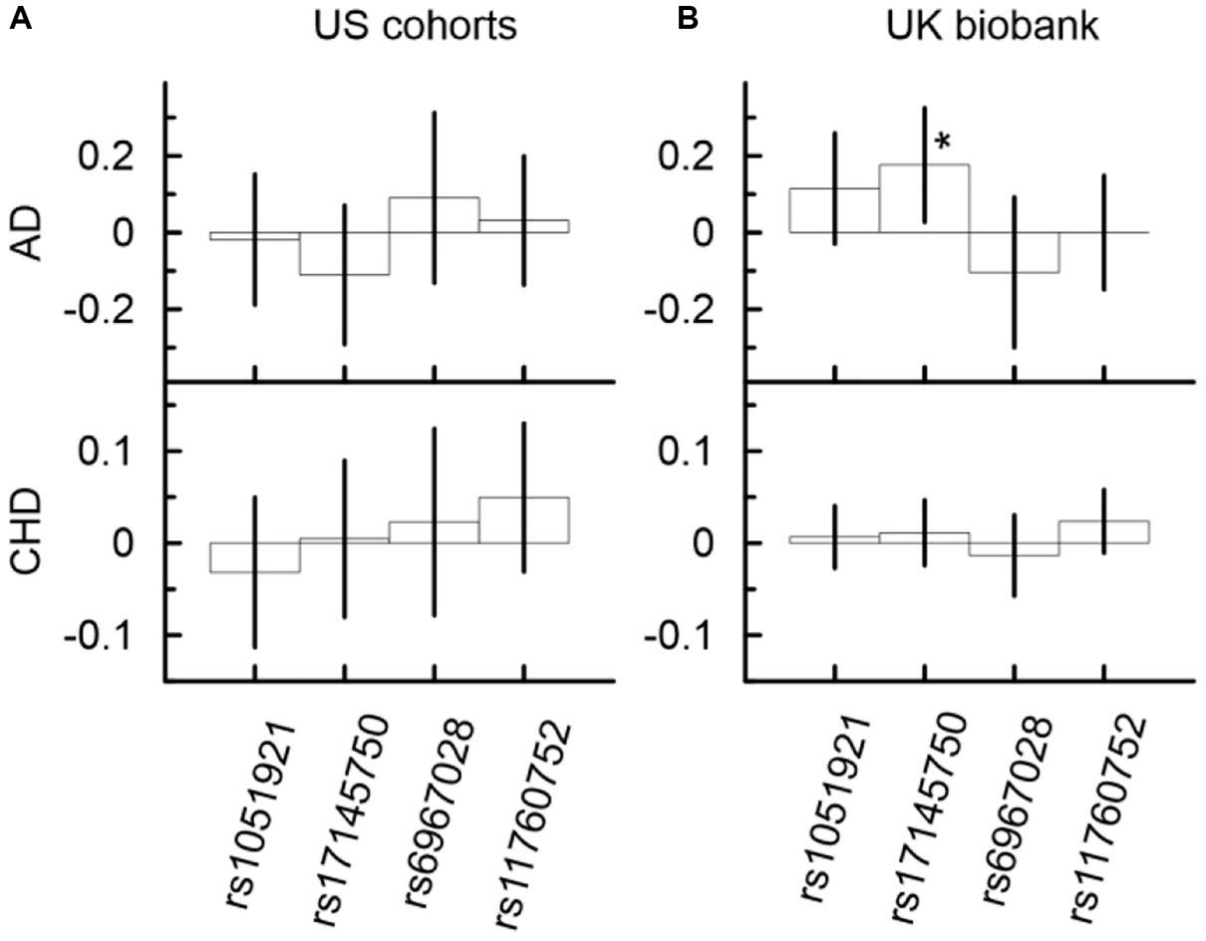
Univariate associations of minor alleles of four SNPs from the *MLXIPL* gene with AD and CHD in two samples drawn from US cohorts (**A**) and UK biobank (**B**). Only one association of rs17145750 with AD was significant in the UK biobank. The Y-axis shows the effect sizes (beta) of the associations of each SNP with the respective outcomes. The X-axis shows SNPs for which association is shown. The vertical solid lines indicate 95% confidence intervals (CIs). Asterisks indicate different levels of significance, i.e., ^*^5 × 10^−4^ ≤ *p* < 0.05.

One SNP (Figure 2, Supplementary Table 6B), rs17145750, demonstrated significant association with AD in the UK Biobank. Its minor allele was significantly associated with higher risk of AD (*β* = 0.18, *p*-value = 2.07 × 10^−2^). In the US meta-analysis, rs17145750 demonstrated a lower AD risk, though it was not significant (*β* = −0.11, *p*-value = 2.34 × 10^−1^). This protective direction of effect was consistent across all US cohorts (Supplementary Figure 2).

Other SNPs (Figure 2, Supplementary Table 6) demonstrated non-significant associations with AD in the UK Biobank and US meta-analysis. Only the minor allele of rs6967028 was associated with significantly increased risk of AD in the LLFS cohort (*β* = 0.53, *p* = 3.23 × 10^−2^).

### Mediation analysis identified significant indirect effects through TG and HDL-C in the associations of SNPs from the *MLXIPL* gene locus with CHD

**Figure 3:**
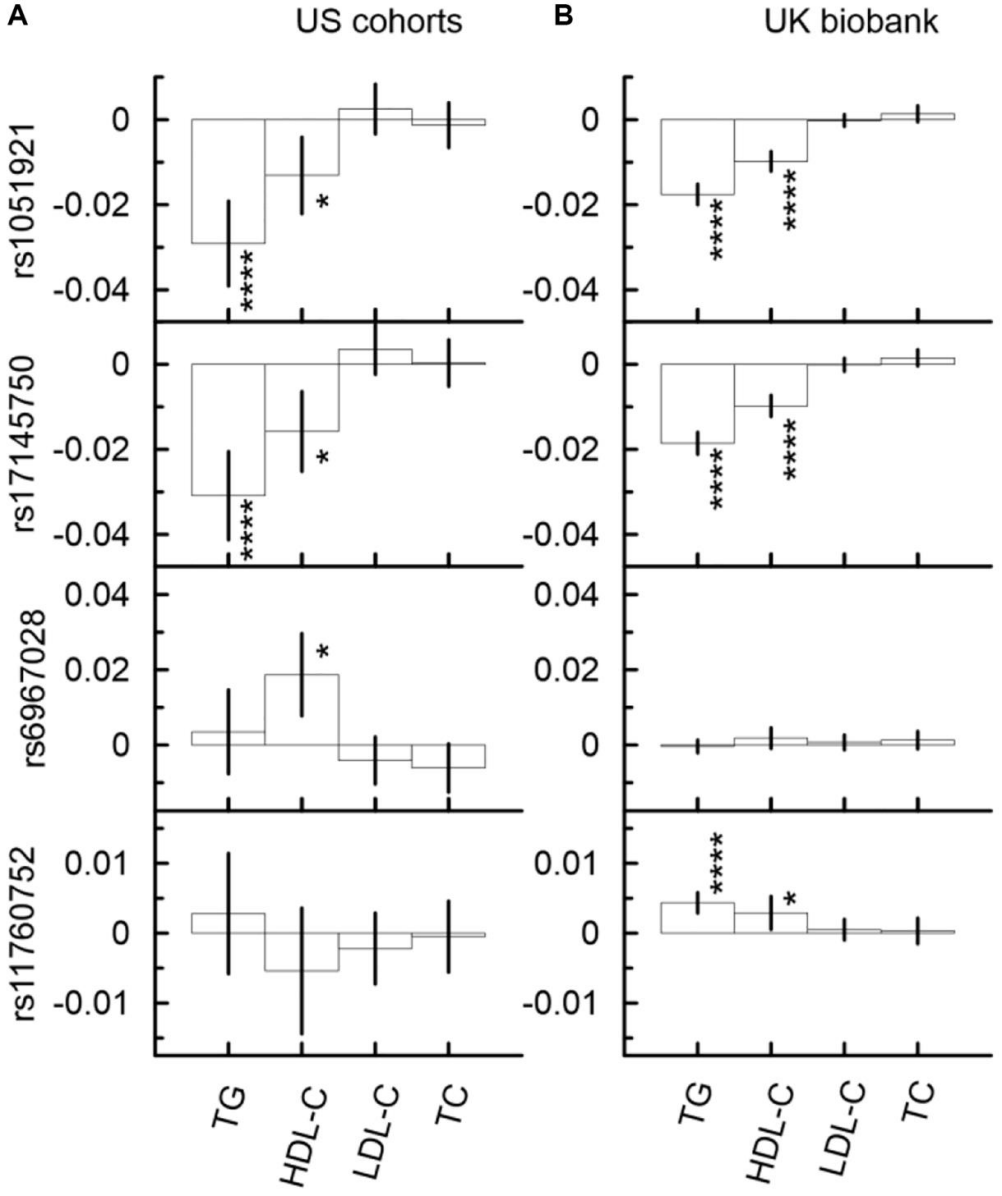
Indirect effects in the associations of the minor alleles of four SNPs from the *MLXIPL* gene with CHD through lipids in two samples drawn from US cohorts (**A**) and UK biobank (**B**). The Y-axis shows the indirect effect sizes (beta) of the associations of respective SNPs with CHD through the mediators depicted on the X axis. The X-axis shows the mediator variable used in the model. The vertical solid lines indicate 95% confidence intervals (CIs). Asterisks indicate different levels of significance, i.e., ^*^5 × 10^−4^ ≤ *p* < 0.05; ^****^p < 5 × 10^−8^.

Although the associations of the considered SNPs with CHD did not attain even the nominal significance in the univariate analysis (Figure 2, Supplementary Table 6), the mediation analysis identified five significant indirect effects in the US meta-analysis and six significant indirect effects in the UKB (Figure 3, and Supplementary Table 6). This analysis showed that the minor allele of rs17145750 was associated with a lower CHD risk through TG (*β*_IE_ = −0.031, *p*_IE_ = 6.46 × 10^−9^) and HDL-C (*β*_IE_ = −0.016, *p*_IE_ = 1.03 × 10^−3^), consistently across all US cohorts except the FHS_C1 (Figure 3A, Supplementary Table 6A, Supplementary Figure 3). The same pattern was observed for rs1051921 (TG: *β*_IE_ = −0.029, *p*_IE_ = 1.13 × 10^−8^; HDL-C: *β*_IE_ = −0.013, *p*_IE_ = 4.09 × 10^−3^). Furthermore, in the UKB cohort (Figure 3B, Supplementary Table 6B) these indirect effects attained GW significant level (TG: *β*_IE_ = −0.019, *p*_IE_ = 5.40 × 10^−44^; HDL-C: *β*_IE_ = −0.010, *p*_IE_ = 3.40 × 10^−14^ for rs17145750; and TG: *β*_IE_ = −0.018, *p*_IE_ = 1.45 × 10^−44^; HDL-C: *β*_IE_ = −0.010, *p*_IE_ = 2.82 × 10^−16^ for rs1051921).

The minor allele of rs6967028 was significantly associated with a higher CHD risk through HDL-C (*β*_IE_ = 0.019, *p*_IE_ = 8.58 × 10^−4^) consistently across all US cohorts (Supplementary Figure 3, Supplementary Table 6A). In the UKB cohort, this indirect effect was of the same sign-direction (*β*_IE_ = 0.002, *p*_IE_ = 1.93 × 10^−1^), but it was non-significant.

### Mediation analysis identified significant indirect effects through TG in the associations of SNPs from the *MLXIPL* gene locus with AD in the UK Biobank

**Figure 4:**
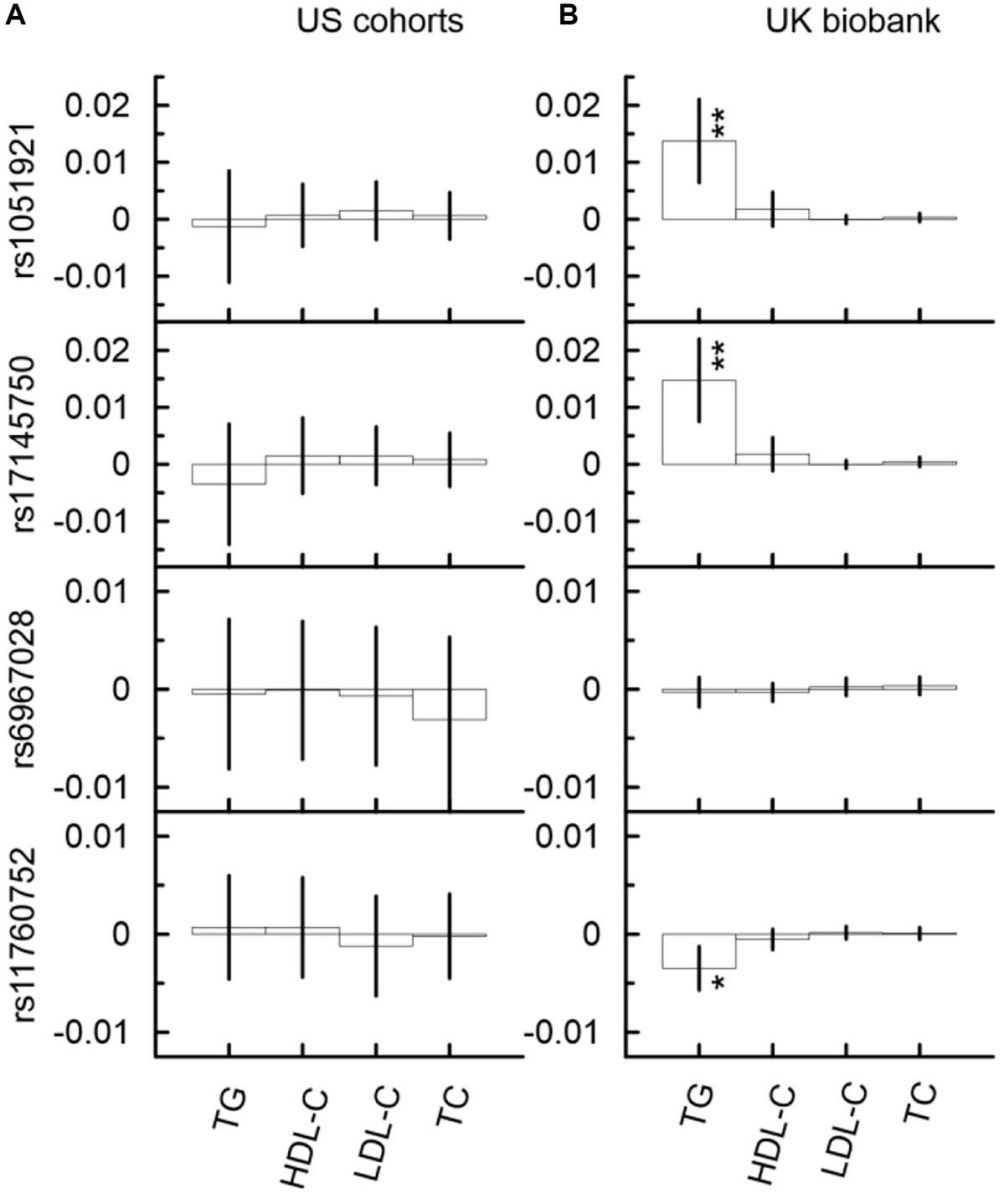
Indirect effects in the associations of the minor alleles of four SNPs from the *MLXIPL* gene with AD through lipids in two samples drawn from US cohorts (**A**) and UK biobank (**B**). The Y-axis shows the indirect effect sizes beta of the associations of respective SNPs with AD through the mediators depicted on the X axis. The X-axis shows the mediator variable used in the model. The vertical solid lines indicate 95% confidence intervals (CIs). Asterisks indicate different levels of significance, i.e., ^*^5 × 10^−4^ ≤ *p* < 0.05; ^**^5 × 10^−6^ ≤ *p* < 5 × 10^−4^.

In the UKB cohort, the mediation analysis identified significant indirect effect through TG in the associations with AD for rs17145750 (Figure 4B, Supplementary Table 6B). This indirect effect was detrimental in the minor allele carriers of rs17145750 (*β*_IE_ = 0.015, *p*_IE_ = 1.90 × 10^−4^). For rs17145750, the indirect effect comprised 8% of the total effect. Also, the direct effect in the association of rs17145750 with AD remained significant in all models (Supplementary Table 6B), i.e., with each of the four considered mediators.

In the US meta-analysis, neither of the selected SNPs demonstrated significant indirect effects in the association with AD in any model of the mediation analysis neither in each cohort separately nor in the meta-sample (Figure 4A, Supplementary Table 6A, Supplementary Figure 4).

## DISCUSSION

This study provides further insight into our understanding of the mechanistic connections between SNPs from the *MLXIPL* gene locus and lipids, as well as TG and HDL-C with AD and CHD, and the heterogeneity in these relationships as discussed below.

### SNPs from the *MLXIPL* gene locus demonstrated independent associations with TG and HDL-C

The results of our unconditional univariate analyses (see Results section) demonstrated that the minor alleles of rs1051921 and rs17145750 were associated with lower levels of TG and TC and higher levels of HDL-C, but were not associated with variations of LDL-C levels in the UK Biobank and US meta-analysis (Figure 1). These two SNPs were in strong LD with rs17145738 and rs3812316, for which GW significant associations with the same traits and the same sign-directions were reported previously [14, 19, 34, 35]. Therefore, our results corroborate previous findings. Additionally, the results of conditional analyses, which are reported in this paper, showed that only 52% of the association of rs1051921 with HDL-C was explained by related variation in TG levels. Also, the conditional analyses demonstrated that the associations of these two SNPs with TC were totally explained by the related variations in TG levels.

The univariate analyses of the associations with lipids distinguished an additional (second) set of SNPs in the *MLXIPL* gene locus. These SNPs demonstrated independent associations as compared to the first set of SNPs mentioned above. In our analyses, the second set had just one member: rs6967028. In contrast to the first set, rs6967028 demonstrated significant associations with TG and HDL-C, but only in the US meta-analysis. Moreover, the leading association was with HDL-C and it was consistent across all cohorts. Only 29% of this association was related to the variation of TG levels, while 53% of its association with TG was explained by the related variation of HDL-C levels. Therefore, our results suggest that there should be biologically specific as well as population specific mechanisms relevant to the observed differences.

VLDL particles as well as their triglycerides and cholesterol content are produced mainly in the liver where MLXIPL is abundantly expressed and controls lipogenesis. The associations of SNPs from the *MLXIPL* gene with HDL-C should be completely mediated through triglycerides, if this were the only organ and the only mechanism which defined levels of TG and HDL-C. In contrast, our analysis demonstrated that only half of the HDL-C associations were explained by the variation of TG levels for SNPs from the first set, rs1051921 and rs17145750. Our results suggest that there should be other mechanisms through which genetic markers from the *MLXIPL* locus can be independently associated with TG and HDL-C levels. These mechanisms can be related to (1) tissue/organ specific/common mechanisms, (2) simultaneous effects in different tissues, and (3) LD of SNPs from the *MLXIPL* and nearby genes. Additionally, the associations with TG and HDL-C can be affected by an exchange of triglycerides and cholesterol between VLDL, LDL, and HDL particles mediated through cholesteryl ester transfer protein (CETP) in the blood stream. Further research is required to identify which, if any, of the above mechanisms are relevant to the observed associations.

The SNPs from the *MLXIPL* gene locus demonstrate complex patterns of associations with TG and HDL-C. Although the *MLXIPL* gene is primarily related to TG synthesis, SNPs from this locus were significantly and independently associated with HDL-C levels. Associations with HDL-C can be affected by several causes as well: (1) microRNA (miRNA) regulation of lipid homeostasis by affecting *MLXIPL* gene expression, (2) packing nascent HDL particles with cholesterol, (3) exchanging TG and cholesterol with VLDL and LDL particles, which is mediated by CETP, and (4) exchanging cholesterol in other tissues. Packing the VLDL particles with triglycerides and cholesterol and filling in the HDL particles with cholesterol are two processes that compete for the same constituents during lipogenesis in the liver. We are suggesting that increasing (decreasing) amounts of triglycerides and consequently cholesterol (considering that the ratio between triglycerides and cholesterol in the VLDL particles is approximately constant (5 to 1)), with which VLDL particles are packed and leave the liver, might lead to decreases (increases) in the amount of cholesterol acquired by HDL particles. Another mechanism is the competing synthesis of triglycerides and cholesterol in the liver, which is initiated by ChREBP (MLXIPL) and sterol regulatory element binding protein (SREBP) and driven by glucose and insulin, respectively. Alternatively, mechanisms in other tissues, e.g., in adipose tissues, which are not related to triglyceride and cholesterol synthesis in the liver, might contribute to the associations of SNPs from the *MLXIPL* gene locus with TG and HDL-C. These alternative mechanisms might be related to the 50% of the association with HDL-C (TG) which was not explained by the mediation effects through TG (HDL-C). The potential effects of the competing processes mentioned above should be further investigated.

### Exogenous exposures shape associations of SNPs from the *MLXIPL* gene locus with TG and HDL-C

Non-overlapping confidence intervals indicate a significant difference in the association of SNPs from the *MLXIPL* gene locus with HDL-C levels in the US vs. UK (see Figure 1, Supplementary Table 5, and Supplementary Figure 1) for both the first set (rs1051921: β_HDL-C_ = 0.98, CI (0.73; 1.23) in the US meta-analysis vs. β_HDL-C_ = 0.51, CI (0.40; 0.62) in the UKB) and the second set (rs6967028: β_HDL-C_ = −0.91, CI (−0.57; −1.25) in the US meta-analysis vs. β_HDL-C_ = −0.10, CI (0.05; −0.26) in the UKB). This difference might be related to different exogeneous exposures in two countries, i.e., differences in climate (weather conditions), socio-economic and historic factors as well as to a difference in food consumption habits. For instance, the amount of high fructose corn syrup (HFCS) consumption per capita in the US is much higher than in the UK [36]. Results from a recent study [37] demonstrated that sugar-sweetened beverage consumption, which is related to the levels of HFCS consumption, may modulate the genetic association of SNPs from the *MLXIPL* gene locus with HDL-C and TG. Our results corroborate those findings. Additionally, in mice with high fat diet, it has been shown that “fructose and glucose supplementation also had distinct effects on expression of the lipogenic transcription factors ChREBP and SREBP-1c” [38]. Elevated consumption of fructose leads to overexpression of SREBP-1c. Therefore, further research is required to identify biologically plausible mechanisms involved in the interaction of SNPs from the *MLXIPL* gene locus with different exogenous exposures.

### SNPs from the *MLXIPL* gene locus demonstrated significant indirect effects in the associations with CHD through TG and HDL-C

Growing evidence demonstrates that elevated triglyceride and decreased HDL-C levels can be independent risk factors for CHD in addition to elevated levels of LDL-C [39]. SNPs from the *MLXIPL* gene locus did not demonstrate significant associations with LDL-C levels and, therefore, they are good candidates to test whether high levels of TG and lower levels of HDL-C are independent risk factors for CHD. Previous studies suggested that TG and HDL-C can be related to CHD risk through different mechanisms. Remnant TG rich particles is one of the possible mechanisms by which TG is related to CHD risk [39]. In addition to reverse cholesterol transport, HDL has many other cardioprotective effects which include anti-oxidative, anti-thrombotic, anti-inflammatory, and anti-apoptotic properties [40].

The univariate analysis of our study did not demonstrate significant associations of SNPs from the *MLXIPL* gene locus with CHD in the US meta-analysis or in the UKB cohort. However, the mediation analysis identified more homogeneous and significant protective indirect effects through TG and HDL-C in the associations of rs1051921 and rs17145750 (SNPs from the first set) with CHD in both US and UKB samples. In the US meta-analysis, the magnitudes of these indirect effects were larger than in the UK Biobank. The difference between the US meta-analysis and UKB was nonsignificant (marginally significant) for the indirect effects through HDL-C (TG). Additionally, a significant detrimental indirect effect through HDL-C was identified in the association of rs6967028 (the second set of SNPs) with CHD in the US meta-analysis but not in the UK Biobank. Moreover, the mediation analysis of CHD risk demonstrated significant differences between the indirect effects through HDL-C in the US and UKB samples, as it follows from non-overlapping CIs (rs6967028: β_CHD_ = −0.91, CI (−0.57; −1.25) in the US meta-analysis vs. β_HDL-C_ = −0.10, CI (0.05; −0.26) in the UKB). Differences in exogenous exposures in the US vs. UK might be a cause of this difference too, which is similar to the difference between the US and UKB samples observed in the associations of these SNPs with TG and HDL-C levels as discussed above.

Our results demonstrate that indirect effects through TG and HDL-C in the associations of SNPs from the *MLXIPL* gene locus with CHD are independent from each other. If they were dependent the indirect effect through TG (β_IE_^TG^ = 0.004, CI (−0.008; 0.015)) in the association of rs6967028 with CHD in the US meta-analysis would be larger than that through HDL-C (β_IE_^HDL-C^ = 0.019, CI (0.008; 0.030)) — which was not observed in our study (Supplementary Table 6 and Figure 2).

These results indicate that there are common and cohort specific mechanisms that link SNPs from the *MLXIPL* gene locus with CHD risk. Moreover, the mediation effects of TG and HDL-C in the associations with CHD are independent from each other.

### SNPs from the *MLXIPL* gene locus demonstrated significant indirect effects in the associations with Alzheimer’s disease through TG in the UK Biobank but not in the US cohorts

A significant detrimental association of rs17145750 with AD in the UK Biobank (β_AD_ = 0.176, CI (0.027; 0.325)) was identified by the unconditional univariate analysis. Additionally, the mediation analysis identified detrimental indirect effects through TG in the associations of rs17145750 (SNP from the first set) and protective indirect effect through TG in the association of rs11760752 with AD in the UK Biobank only. However, this indirect effect was small comprising 8% of the total effect. Also, the direct effect in the association of rs17145750 with AD was significant. No significant effects were detected in the US meta-analysis — neither total, nor direct, nor indirect effects.

These results suggest cohort specific mediation effects that involve TG related mechanisms in the UKB cohort. Our results suggest that increased TG levels might be protective against AD. This idea is supported by some previous studies [28, 41, 42], but other studies reported the opposite effects of TG [4, 43]. Possible detrimental and protective TG related effects/mechanisms have been already highlighted in previous studies. Increased TG levels in midlife were associated with heavier Aβ and tau pathology in the brain [44, 45]. However, high levels of TG can demonstrate protective effects on cognitive functions at older age because they can increase the transport of ghrelin and insulin through the blood-brain barrier, affect the expression of orexigenic hypothalamic peptides; and because unsaturated fatty acids can decrease production of inflammatory cytokines [46–51]. Considering the numerous mechanisms by which TG can affect the health of the human brain, further research using more advanced statistical methods/approaches and larger or more homogeneous samples is required to clarify the cause of this heterogeneity, conditions under which protective and detrimental effects are prevailing, and ultimately to resolve the puzzle about the contribution of TG to AD risk.

Considering the common ancestry of the US and UK cohorts of this study, one can hypothesize that the difference in the associations with AD might be caused by a difference in exogenous exposures (external environment, socio-economic differences between two countries, etc.) through the individual’s life course, which shapes TG related mechanisms. Further research is required for distinguishing those exogenous factors and related biological processes and mechanisms.

In conclusion, the results demonstrated independent associations of SNPs from the *MLXIPL* gene locus with TG and HDL-C. SNPs from one set (represented by rs1051921 and rs17145750) demonstrated stronger association with TG, while SNPs from another set (represented by rs6967028) demonstrated stronger association with HDL-C. Neither associations with TG nor with HDL-C were totally explained by one another, i.e., HDL-C and TG, respectively, in either set of SNPs. This suggests independent or overlapping mechanisms/pathways in the regulation of TG and HDL-C by genetic markers from the *MLXIPL* gene locus. Further research is required to identify the cause of this independence, i.e., for instance, whether it can be attributed to regulatory functions, interactions with or independent effects of nearby genes in this locus, or isoform difference of related proteins.

Our findings highlight significant differences in the magnitudes of the genetic associations with HDL-C in the UK and US samples. Previous publications indicated that such differences can be attributed to differences in exogenous exposures, for example, in consumption of fructose. Our results corroborate those findings.

Accumulating evidence suggests that high levels of TG and low levels of HDL-C are additional risk factors for CHD, and their effects are independent from those caused by high levels of LDL-C. Our results support this hypothesis by demonstrating significant and independent mediation effects through TG and HDL-C on CHD risk. Additionally, the results of this study highlight significant indirect associations of SNPs from the *MLXIPL* gene locus with AD risk through TG related mechanisms in the UK cohort, but not in the US-based cohorts. Further research can help to identify plausible biological mechanisms of this association.

The results of this paper suggest that there is a trade-off associated with TG related mechanisms in the effects of SNPs from the first set, rs1051921 and rs17145750. In the UK cohort, carriers of their minor alleles are at a lower risk of CHD and at a higher risk of AD. No such trade-off was observed in the US meta-analysis, where significant associations were observed with CHD only. Therefore, the results suggest that the trade-off might be related to modifiable factors shaped by exogenous exposures, which are different in the US and UK. Meanwhile, other exogenous exposures can cause the differences reported in this paper.

Finally, the results of this study suggest that genetic associations of SNPs from the *MLXIPL* gene locus with lipids, AD, and CHD are shaped by exogenous exposures. Further study of the related biological mechanisms can help to elucidate the related, modifiable risk factors.

### Limitations

One limitation of this study is that the significance levels of the reported results were not corrected according to the number of tests performed. Another limitation is that no corrections for familial structures were made in the mediation analyses and in-house R-code was used, but not R-packages.

## METHODS

### Accession numbers

This manuscript was prepared using limited access data obtained though dbGaP from the ARIC (phs000280.v5.p1), CHS (phs000287.v5.p1), FHS (phs000007.v31.p12), MESA (phs000209.v13.p3), and WHI (phs000200.v10.p3) studies. The LLFS data (release from Oct 2020) were available from the LLFS Data Management and Coordinating Center (Washington University, St. Louis, USA). This research was also conducted using data from UK Biobank (release from December 2020), a major biomedical database (http://www.ukbiobank.ac.uk/).

### Study cohorts

Data for the US sample available for this study were obtained from seven longitudinal US population/community-based cohorts, which included the Atherosclerosis Risk in Communities (ARIC) study [52], Cardiovascular Health Study (CHS) [53], Framingham Heart Study (FHS_C1 and FHS_C2 cohorts, see definition below) [54], Multi-Ethnic Study of Atherosclerosis (MESA) [55], the Genomics and Randomized Trials Network (GARNET) sub-study of the Women’s Health Initiative (WHI) [56, 57], and the Long Life Family Study (LLFS) [58–60]. In addition to the U.S., the LLFS also includes participants from Denmark. Data for UK sample were drawn from the UK Biobank (UKB) [61, 62]. Only data on individuals of European ancestry were considered in the analyses. The FHS parental (FHS_C1) and offspring (FHS_C2) cohorts were analyzed separately, as they included participants from different generations. The FHS grandchildren cohort, 3rd generation, was excluded from the analyses because of the younger ages of the participants. Basic demographic information for the genotyped participants in the selected studies is provided in Supplementary Table 1 (Supplementary Data).

### Genotypes

We considered directly genotyped SNPs that were available from the same customized Illumina CVDSNP55v1_A chip (50K SNPs) in five cohorts (ARIC, CHS, FHS_C1, FHS_C2, and MESA); Illumina HumanOmni 2.5 Quad chip (2.5M SNPs) in LLFS, Illumina HumanOmni1-Quad_v1-0_B chip (1M SNPs) in WHI (GARNET), and the UK Biobank Axiom Array (850K SNPs) in the UK Biobank. Within the chromosome region associated with the *MLXIPL* gene (including exon and intron regions as well as 5Kb flanking regions on both sides, see Supplementary Figure 5), hereafter referred to as the *MLXIPL* gene locus, there were 6 SNPs present on all arrays with minor allele frequency (MAF) greater than 5% in all cohorts. Analysis of the linkage disequilibrium (LD) structure of these genotyped SNPs was performed for unrelated individuals by using plink (version 1.9) software [63, 64]. It identified three independent sets of SNPs with the LD between SNPs from different sets being smaller than |r|^2^ < 0.25, which was consistent in all cohorts. Four SNPs (Supplementary Tables 2 and 3, Supplementary Data) were selected to represent three sets of SNPs from the *MLXIPL* gene locus. We included two SNPs, rs1051921 and rs17145750, in the first set because these SNPs demonstrated comparable significance (*p*-values) of the associations with TG and HDL-C. The LD between the two SNPs is moderate (not too high) and varied across the cohorts (0.73 < r^2^ < 0.80), see Supplementary Table 3. For the second and the third sets of SNPs, we included rs11760752 and rs6967028, respectively, as they demonstrated the most significant associations with TG and/or HDL-C in each set. All four selected SNPs in all considered cohorts were in Hardy-Weinberg equilibrium (HWE) with the HWE exact test *p*-values > 0.01.

Information on the selected SNPs and their study-specific minor allele frequencies is presented in Supplementary Table 2 (Supplementary Data). The minor allele frequencies were consistent across all cohorts. The selected SNPs, except rs1051921 and rs17145750 (0.73 < r^2^ < 0.81), had low LD (0.006 < r^2^ < 0.057), which was consistent across all cohorts (Supplementary Table 3, Supplementary Data).

### Phenotypes

AD was defined based on diagnoses made according to the National Institute of Neurological and Communicative Disorders and Stroke and the Alzheimer’s disease and Related Disorders Association in the FHS cohorts; based on ICD-9 (331.0x) codes in Medicare service use files in the CHS study; based on ICD-9 and ICD-10 (G30, F00) codes in the UK Biobank and interviews with participants or their proxies (self-reports) in the LLFS. Information on AD was not available in the ARIC, MESA, and WHI cohorts. Data on coronary heart disease (CHD) was available from examinations, clinic visits, hospital and physician records, and interviews with participants or their proxies (self-reports) in all cohorts. Information on the number of incident diseases is given in Supplementary Table 1 (Supplementary Data).

Four continuous phenotypes, HDL-C (mg/dL), TG (mg/dL), LDL-C (mg/dL), and TC (mg/dL) with pair-wise correlations ranging from *r*~ –0.42 for HDL-C and TG to *r*~0.95 for LDL-C and TC (as it was observed in the largest sample of the UKB) were considered (Supplementary Table 4, Supplementary Data). They were measured multiple times during the follow-up of the same individuals in all seven longitudinal cohorts. In the UK Biobank, longitudinal information was available for a small number of participants and, therefore, we selected only one (last) examination. Blood lipids were measured from fasting venous blood samples. Standard enzymatic methods were used for directly measuring HDL-C, TC, and TG. LDL-C was directly measured in the UKB and calculated by using the Friedwald formula in other studies. In all analyses throughout this paper, we used measurements of quantitative (lipid) phenotypes taken before the onsets of CHD and AD. In the univariate analyses for the lipid outcomes, we used all these measurements. In the mediation analyses, we selected measurements based on the latest examination at which all lipid measurements were available for the same individual (Supplementary Table 1, Supplementary Data) (see also the Statistical Analyses Section).

### Statistical analyses

Our analysis was implemented as a synthesis of (i) the traditional univariate (unconditional and conditional) analysis of the selected lipid traits, and (ii) mediation analysis (see below). This approach allowed us to identify more homogeneous mechanistic relationships between lipids, AD, CHD, and tag SNPs from the MLXIPL gene locus and delineate the effects of exogenous exposures.

Data on individuals with complete information on four SNPs, and four quantitative traits, HDL-C, LDL-C, TG, and TC, measured at all examinations before the onset of CHD and AD were used in the univariate analyses with quantitative outcomes. The measurements of the lipid traits at the last examination (but before the onset of the two diseases) were used when considering AD and CHD as the outcomes. We performed three types of analyses – univariate unconditional, conditional, and mediation – in each cohort separately to examine relationships between four SNPs from the *MLXIPL* gene and these phenotypes. We combined statistics across cohorts using a fixed-effect model with inverse-variance weighting (METAL software [65]). Results for the US meta-analysis were compared to the corresponding results from the UKB.

The univariate analyses in all cohorts were conducted using the generalized estimating equation (GEE) [66, 67] (gee package, version 4.13–23, in R) with unstructured correlation matrix for linear models with continuous outcomes (TG, HDL-C, LDL-C, and TC). The Cox proportional hazards model (coxph functions in R) was used for the time-to-event outcomes (AD and CHD) with age used as a time variable (no left truncation). This approach allowed us to correct for familial structure by using family number as a cluster variable in the FHS and LLFS cohorts. All univariate analyses of lipid traits were performed by using additive genetic models with the minor allele of each SNP considered as an effect allele. We evaluated the associations of four selected SNPs with each lipid trait, given their measurements at all available examinations (before the onset of CHD and AD), as defined above. All models in all analyses in this paper were adjusted for sex, birth year, and the data collection site (available in ARIC, CHS, MESA, WHI, and LLFS), and age at examination for continuous phenotypes (herein referred to as basic adjustments).

The conditional analyses were an extension of the univariate unconditional analyses when, in addition to the basic adjustments, the models were also adjusted for one of the remaining continuous phenotypes. These analyses were performed for the continuous outcomes only. In this case, each continuous phenotype other than the continuous outcome was used as a covariate. We used the simplest approach by considering pair-wise combinations only. For example, we evaluated associations of the rs17145750 with TG using three models with adjustment for HDL-C, LDL-C, or TC. Therefore, for each SNP, univariate (unconditional and conditional) analyses provided the results from 16 models for four continuous traits. Additionally, we considered models with all possible pairs of SNPs included. This analysis provided 12 (=6/2 × 4) additional betas for each SNP from 6 models for four lipid traits. After multiple testing (28 tests) *p_MT_* = 1.79 × 10^−3^ (=0.05/28) is considered as a threshold for statistical significance in multiple tests for qualitative (lipids) traits in the US meta-analysis.

Mediation analyses were performed to investigate mutual mediation roles of the selected continuous phenotypes in the associations of four selected SNPs with AD and CHD in models with basic adjustment. These analyses evaluated natural indirect effects mediated by an examined continuous phenotype and natural direct effects, which can be related to the direct effects of the considered alleles or through other unspecified mechanisms [29, 30]. For the disease (time-to-event) outcomes, mediation analysis was performed using continuous phenotypes as mediators measured at the last examination that preceded the onset of both diseases. We compared heterozygous and minor homozygous genotypes combined versus major homozygous genotypes. Heterozygous and minor homozygous genotypes were combined in order to increase statistical power of the analysis. We assumed that there were no other unmeasured confounders between the assignment of a mediator and a disease. Marginal structural models were used for estimating direct and indirect effects for the time-to-event outcomes and were conducted by using in-house R-code developed based on published literature [29–33]. Linear regression models were adopted for continuous mediators and Cox proportional hazard models were used for the time-to-even outcomes. Robust standard errors were obtained using a boot-strapping method with 1000 replicates. In the mediation analyses, no corrections for familial structures were used.

### Data availability

This manuscript was prepared using limited access data obtained though dbGaP ARIC (phs000280.v5.p1), CHS (phs000287.v5.p1), FHS (phs000007.v31.p12), MESA (phs000209.v13.p3), and WHI (phs000200.v10.p3) studies. The LLFS data were provided by the LLFS Data Management and Coordinating Center (Washington University, St. Louis, USA). Phenotypic and genetic datasets of the UK Biobank are available through the following link: http://www.ukbiobank.ac.uk/.

## Supplementary Materials

Supplementary Acknowledgment

Supplementary Figures

Supplementary Table 1

Supplementary Table 2

Supplementary Table 3

Supplementary Table 4

Supplementary Table 5A

Supplementary Table 5B

Supplementary Table 5C

Supplementary Table 5D

Supplementary Table 6A

Supplementary Table 6B
