# Supplementary material for: Exogenous exposures shape genetic predisposition to lipids, Alzheimer’s, and coronary heart disease in the MLXIPL gene locus
Source: Aging (Albany NY). 2023 Apr 18;15(9):3249–72. doi: 10.18632/aging.204665 (PMC10449285; doi:10.18632/aging.204665)
Supplement: Supplementary Figures [file aging-15-204665-s002.pdf]

SUPPLEMENTARY FIGURES

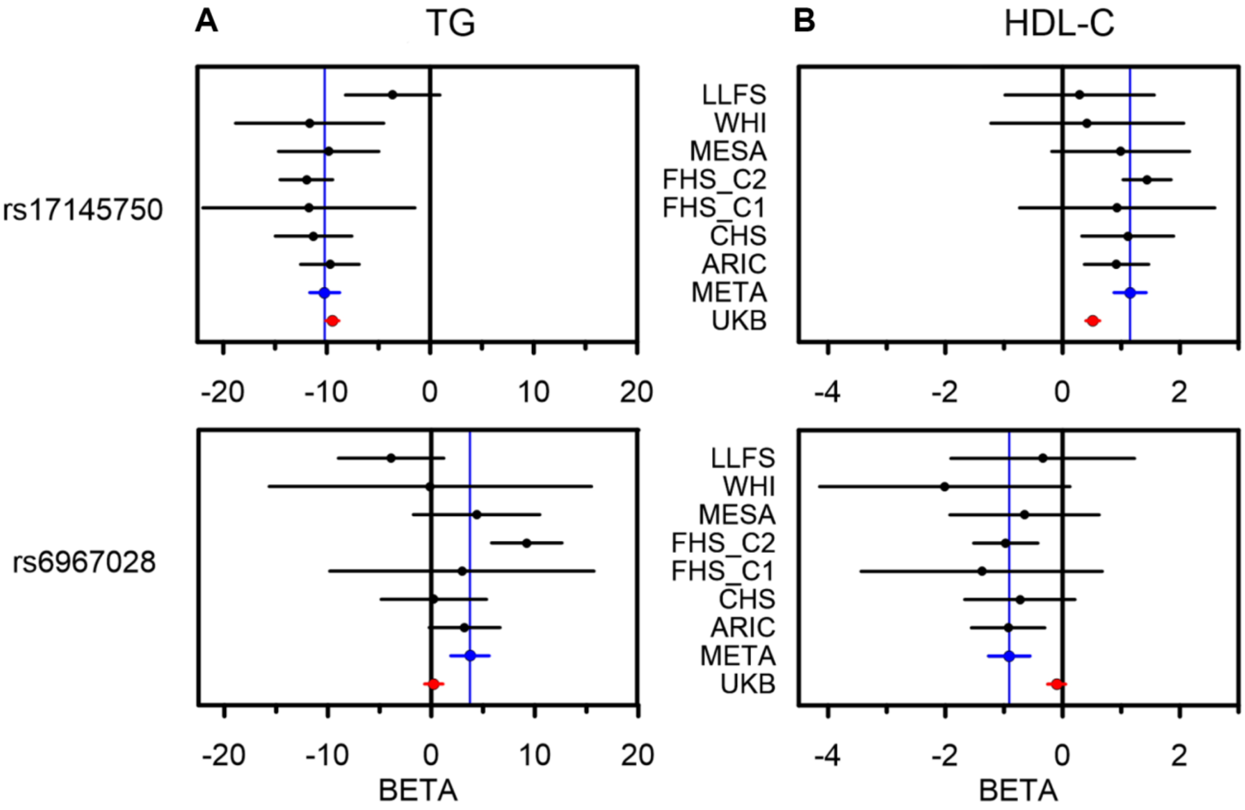

Supplementary Figure 1. Associations of rs17145750 and rs6967028 from the MLXIPL gene locus with TG and HDL-C in each cohort separately. The results are presented for cohorts included in the meta-analysis (black dots), the meta-analysis itself (META, blue dots), and UKB (red dots) for comparison. The X-axis shows the effect sizes beta of the associations with the outcomes: (**A**) TG and (**B**) HDL-C. The horizontal lines indicate 95% confidence intervals. Blue vertical lines represent the effect sizes in the US meta-analysis.

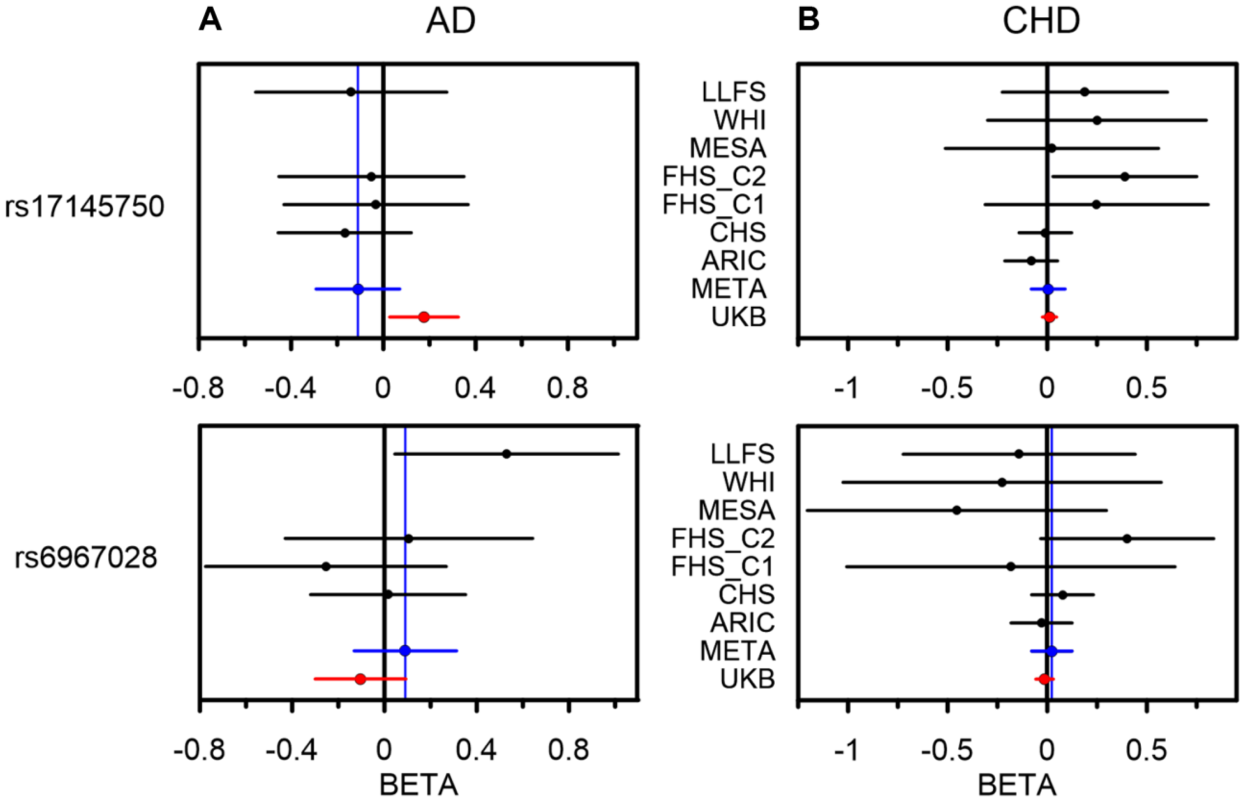

Supplementary Figure 2. Associations of rs17145750 and rs6967028 from the *MLXIPL* gene locus with AD and CHD in each cohort separately. The results are presented for cohorts included in the meta-analysis (black dots), the meta-analysis itself (META, blue dots), and UKB (red dots) for comparison. The X-axis shows the effect sizes beta of the associations with the outcomes: (**A**) AD and (**B**) CHD. The horizontal lines indicate 95% confidence intervals. Blue vertical lines represent the effect sizes in the US meta-analysis.

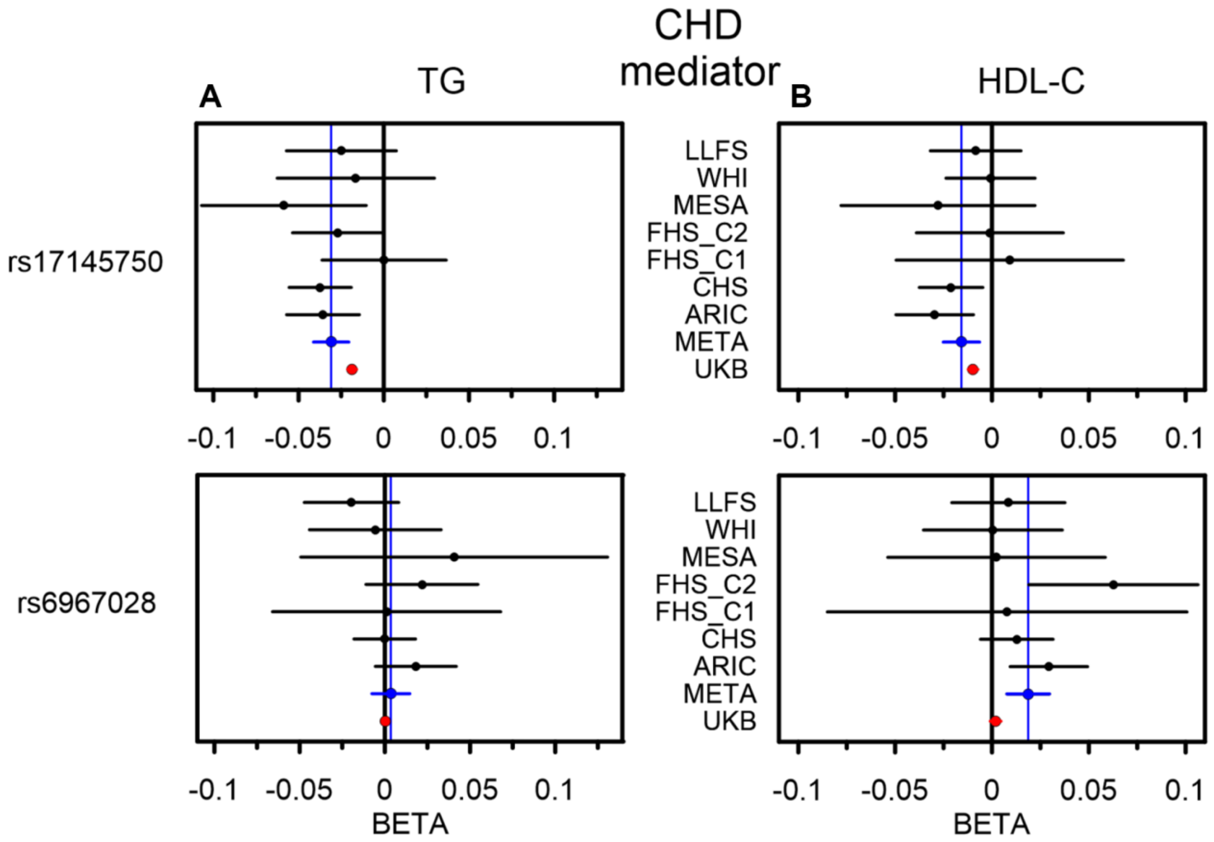

Supplementary Figure 3. Indirect effect through TG and HDL-C in the associations of rs17145750 and rs6967028 from the *MLXIPL* gene locus with CHD in each cohort separately. The results are presented for cohorts included in the meta-analysis (black dots), the meta-analysis itself (META, blue dots), and UKB (red dots) for comparison. The X-axis shows the effect sizes beta of the indirect effect (IE) through (**A**) TG and (**B**) HDL-C in the associations with CHD. The horizontal lines indicate 95% confidence intervals. Blue vertical lines represent the effect sizes in the US meta-analysis.

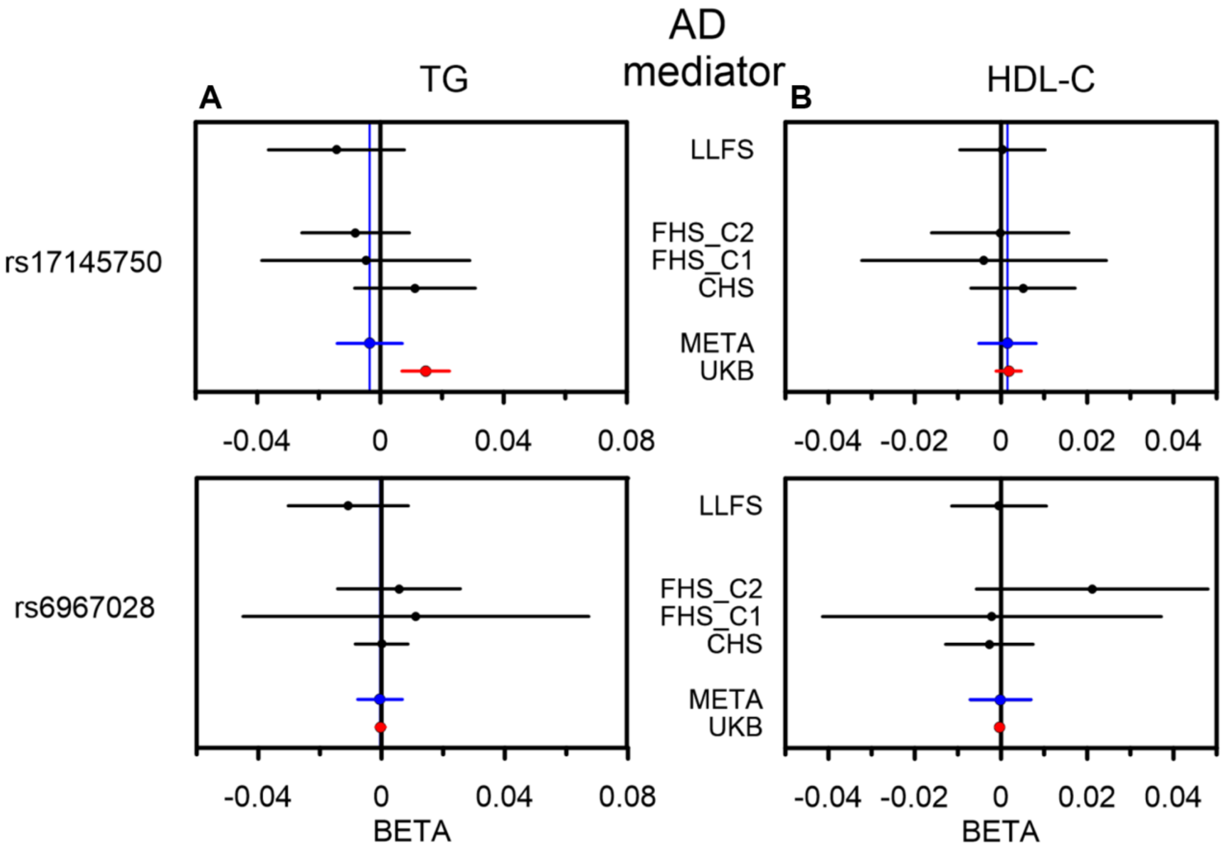

Supplementary Figure 4. Indirect effect through TG and HDL-C in the associations of rs17145750 and rs6967028 from the *MLXIPL* gene locus with AD in each cohort separately. The results are presented for cohorts included in the meta-analysis (black dots), the meta-analysis itself (META, blue dots), and UKB (red dots) for comparison. The X-axis shows the effect sizes beta of the indirect effect (IE) through (**A**) TG and (**B**) HDL-C in the associations with AD. The horizontal lines indicate 95% confidence intervals. Blue vertical lines represent the effect sizes in the US meta-analysis.

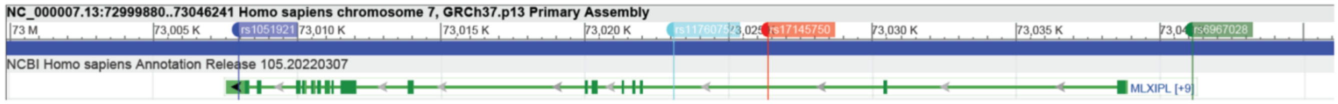

Supplementary Figure 5. Position of four selected SNPs in the *MLXIPL* gene region. (From dbSNP, NCBI).
